# Peroxiredoxin 6 mediates the protective function of curcumin pretreatment in acute lung injury induced by serum from patients undergoing one-lung ventilation in vitro

**DOI:** 10.1186/s12890-022-01988-y

**Published:** 2022-05-12

**Authors:** Hui-Ting Li, Fang Tan, Tian-Hua Zhang, Long-Hui Cao, Hong-Ying Tan, Wen-Qian Lin, Wei-An Zeng, Xin-Jin Chi

**Affiliations:** 1 Department of Anesthesiology, State Key Laboratory of Oncology in Southern China, Collaborative Innovation Center for Cancer Medicine, Sun Yat-Sen University Cancer Center, Guangzhou, 510060 China; 2 Department of Anesthesiology, The Seventh Affiliated Hospital of Sun Yat-Sen University, Shenzhen, 518107 China; 3 Department of Anesthesiology, The Third Affiliated Hospital of Sun Yat-Sen University, Guangzhou, 510635 China

**Keywords:** One-lung ventilation, Lung injury, Curcumin, Peroxiredoxin 6, NF-κB signaling

## Abstract

**Background:**

Curcumin has attracted much attention due to its wide range of therapeutic effects. In this study, we used serum collected from patients undergoing one-lung ventilation (OLV) to establish an in vitro acute lung injury (ALI) model to explore the potential protective mechanism of curcumin on ALI. Our study provides a new reference for the prevention and treatment of ALI induced by OLV.

**Methods:**

A549 cells were treated with 20% serum from patients undergoing OLV to establish an in vitro ALI model. Curcumin, at a dose of 40 μg/ml, was administered two hours prior to this model. The levels of inflammation and oxidative stress markers were observed by Western blot, qRT–PCR, ELISA and reactive oxygen species assay. Additionally, the expression of peroxiredoxin 6 (Prdx6) and proteins involved in the NF-κB signaling pathway was evaluated.

**Results:**

Twenty percent of serum collected from patients undergoing OLV downregulated the expression of Prdx6, leading to the activation of the NF-κB signaling pathway, which was associated with the subsequent overproduction of inflammatory cytokines and reactive oxygen species. Pretreatment with curcumin restored Prdx6 downregulation and inhibited NF-κB pathway activation by suppressing the nuclear translocation of P65, eventually reducing inflammation and oxidative stress damage in A549 cells.

**Conclusions:**

Prdx6 mediated the protective function of curcumin by inhibiting the activation of the NF-κB pathway in ALI in vitro.

## Background

One-lung ventilation (OLV) often results in mechanical ventilator-related lung injuries, which poses a great challenge to pulmonary function in patients undergoing chest surgery. The main causes of lung surgery-related mortalities have shifted from cardiac and surgical complications to pulmonary complications such as acute lung injury (ALI) [1]. Unlike other complications, the rate of ALI has not declined over the past decades [2, 3]. It is worth noting that pneumonectomy is associated with an increased risk of lung injury, and minor excision thoracic surgery may also result in similar pathology [4]. During thoracic surgery, the combined impacts of surgical procedures and OLV can lead to the release of pro-inflammatory factors and pro-oxidants that contribute to the development of ALI [5, 6]. Moreover, plasma collected from patients undergoing lobectomy or pneumonectomy has also shown evidence of oxidative damage [7]. Although the incidence of ALI caused by OLV remains high, there are currently no effective prevention methods. Therefore, it is particularly crucial to find effective therapeutic drugs.

Curcumin [1,7-bis(4-hydroxy-3-methoxyphenyl)-1,6-heptadiene-3,5-dione], a naturally occurring polyphenol extracted from the rhizome of turmeric, has gained much attention because of its pleiotropic pharmacological properties, such as anti-inflammatory [8], antioxidant [9], hypoglycemic [10], antibacterial [11] and immunomodulatory activities [12]. Newly synthesized metal-curcumin complexes have been shown to increase the solubility, cellular uptake and bioavailability of curcumin, improving its anti-inflammatory, antioxidant, antibacterial and antiviral effects and enhancing its clinical applications [13, 14]. Several studies have confirmed that curcumin has a protective effect on lung injury caused by a variety of pathological states that involve multiple regulatory mechanisms [15–17]. However, there are few studies on the role of curcumin in ALI induced by OLV and thoracic surgery.

Peroxiredoxin 6 (Prdx6) is the only mammalian 1-Cys member of the peroxiredoxin family. It has many functions, such as glutathione peroxidase (Gpx) activity, calcium-independent phospholipase A2 (aiPLA2) activity, and lysophosphatidylcholine acyltransferase (LPCAT) activity. It is involved in redox homeostasis, phospholipid turnover, glycolipid metabolism, and cell signal transduction. Existing research has indicated that Prdx6 is closely related to the occurrence and development of ALI. In the lipopolysaccharide (LPS)-induced lung injury mouse model, inactivation of the aiPLA2 activity of Prdx6 was associated with reduced mortality and prevention of lung inflammation and oxidative stress [18]. Similarly, Yang et al. found that deletion of Prdx6 exaggerated LPS-induced ALI with increased oxidative stress in vivo [19]. These results suggest that Prdx6 may play an important role in ALI induced by OLV, and further studies are needed to test our hypothesis.

In the present study, we first established an in vitro model of ALI induced by the intervention of serum collected from patients undergoing OLV and then used this model to explore the effect of pretreatment with curcumin. Finally, we explored the underlying regulatory mechanisms involved.

## Materials and methods

### Patients and methods

From February 1, 2021, patients who underwent video-assisted thoracoscopic surgery (VATS) for lung cancer at the Sun Yat-Sen University Cancer Center (SYSUCC; Guangzhou, China) were assessed for eligibility. Exclusion criteria included pneumonectomy and thoracotomy. Written informed consent was obtained from all patients. The study protocol was approved by the ethics committee of SYSUCC.

### Anesthetic procedure, surgery and blood sample collection

Anesthetic procedures were standardized for all patients to minimize the impact of anesthesia differences between patients, with the use of double-lumen bronchial catheterization and positioning with fiberoptic bronchoscopy to ensure OLV. Intravenous anesthesia induction was applied using bolus doses of fentanyl, cisatracurium and propofol. Intravenous inhalation combined with anesthesia was applied intraoperatively using continuous infusions of remifentanil, cisatracurium and propofol and continuous inhalation of sevoflurane.

A protective ventilation strategy was applied during OLV. Briefly, a low tidal volume of ~ 6–7 ml/kg body weight and a 3–5 cm H_2_O of positive end-expiratory pressure were applied, with the peak airway pressure limited below 30 cm H_2_O. The concentration of inhaled oxygen in the ventilated lung fluctuated from 80 to 100% and was adjusted according to repeated arterial blood gas analysis. If pulse oxygen saturation (SpO_2_) fell below 80%, then high-frequency ventilation equipment was used. VATS-assisted lobectomy was performed by three thoracic surgeons in the same medical group using similar surgical techniques. Central venous blood samples were obtained from the internal jugular vein before OLV and before the end of OLV and were marked as OLV_before_ and OLV_after_. After placement for 30 min at room temperature, the entire blood sample was centrifuged at 3000 bpm for 15 min. Then, the serum in the upper layer was collected and was preserved at − 80 °C. The OLV_before_ and OLV_after_ serum of all patients were merged respectively and the pooled serum was aliquoted and used for the following experiments.

## Cells and reagents

The human alveolar epithelial cell line A549 was cultured in Dulbecco’s S modified Eagle’s medium (DMEM, Gibco, USA) containing 10% fetal bovine serum (FBS, HyClone, USA) and 1% penicillin–streptomycin (Thermo Fisher Scientific, USA). The cells were incubated at 37 °C in a humidified 5% CO_2_ incubator. Curcumin (78246-100 mg, Sigma–Aldrich, USA) was dissolved in dimethyl sulfoxide (DMSO, D2650-100 mL, Sigma–Aldrich, USA) and diluted with a complete medium. Small interfering RNA (siRNA) targeting Prdx6 and scrambled RNA (NC-siRNA) controls were purchased from Kidan Biosciences (Guangzhou, China). The sequence and antisequence of Prdx6 siRNA-1, siRNA-2 and siRNA-3 were 5′-GACAGUGUUGAGGACCAUCUUTT-3′ and 5′-AAGAUGGUCCUCAACACUGUCTT-3′; 5′-CCGAAAGGAGUCUUCACCAAATT-3′ and 5′-UUUGGUGAAGACUCCUUUCGGTT-3′; and 5′-CGCAUCCGUUUCCACGACUUUTT-3′ and 5′-AAAGUCGUGGAAACGGAUGCGTT-3′, respectively. The transfection process was carried out using Lipofectamine 3000 reagent (Carlsbad, Invitrogen, USA) according to the manufacturer’s instructions.

## Experimental design

### Serum concentrations from patients undergoing OLV induce lung inflammation and oxidative stress

To verify whether the serum from patients undergoing OLV could induce lung injury and to explore the appropriate concentration of OLV serum, the cells were treated with FBS-free DMEM with or without serum from patients undergoing OLV for 48 h when A549 cells reached 70–80% confluence. The groups were set as follows: (1) control group: only the same volume of FBS-free DMEM was added, (2) 10% OLV_before_ serum: FBS-free DMEM containing 10% serum from patients before OLV, (3) 20% OLV_before_ serum: FBS-free DMEM containing 20% serum from patients before OLV, (4) 30% OLV_before_ serum: FBS-free DMEM containing 30% serum from patients before OLV, (5) 10% OLV_after_ serum: FBS-free DMEM containing 10% serum from patients after OLV, (6) 20% OLV_after_ serum: FBS-free DMEM containing 20% serum from patients after OLV, and (7) 30% OLV_after_ serum: FBS-free DMEM containing 30% serum from patients after OLV.

After 48 h of intervention, cell morphology was observed with an electric fluorescence microscope (Nikon ECLIPSE Ti2, Nikon, Japan). The expression of IL-6 at the protein and gene levels was detected by Western blot and qRT–PCR (quantitative reverse transcription PCR) in A549 cells. In addition, to test reactive oxygen species (ROS) levels, a ROS assay was performed using dichlorodihydrofluorescein diacetate (DCFH-DA) staining according to the manufacturer’s instructions.

### Dosage of curcumin for reducing OLV_after_ serum-induced lung injury

In our preliminary work, the proliferation activity of curcumin on A549 cells was detected by using MTS. We analyzed the IC50 value (91.22 μg/ml) of curcumin in A549 cells for 2 h. Therefore, we selected two doses lower and a dose greater than IC50 (40, 80 and 160 μg/ml) as experimental concentrations to explore the appropriate dose of curcumin, which can affect OLV serum-induced lung injury without harming the cells. We first preprocessed the A549 cells with three different concentrations (40, 80 and 160 μg/ml) of curcumin for 2 h. Curcumin was dissolved in DMSO on the day of the experiment and diluted in a serum-free medium to ensure that the final concentration of DMSO was < 0.05%, and the same concentration of DMSO was used in the control group. Then, according to the results based on the previous sections, 20% OLV_after_ serum (20% serum from patients after OLV) was added to induce lung inflammation and oxidative stress injury. After the cells were treated with 20% OLV_after_ serum for 48 h, the expression of IL-6 and the level of ROS were assessed by Western blot, qRT–PCR and DCFH-DA staining.

### The effect of curcumin pretreatment on OLV_after_ serum-induced inflammation and oxidative stress injury in A549 cells

Here, we pretreated A549 cells with 40 μg/ml curcumin (the dose of curcumin was determined according to the results obtained in the previous section) for 2 h. Then, after 2 h, the culture medium containing curcumin was discarded, and the cell surface was thoroughly washed three times with PBS. Next, the A549 cells were incubated with FBS-free DMEM containing 20% serum from patients after OLV for 48 h. The cells and cell culture medium were harvested for subsequent inflammation and oxidative stress factor measurements. ROS was detected by DCFH-DA staining. Meanwhile, the expression of Prdx6 was tested by qRT–PCR, Western blot and immunofluorescence.

### The role of Prdx6 in the protective effects of curcumin pretreatment on serum from OLV patient-induced lung injury and its related mechanism

To explore the potential mechanism of the protective effect of curcumin, the expression of proteins involved in the NF-κB signaling pathway was analyzed. To verify the relationship between Prdx6 and the NF-κB signaling pathway in vitro, we used siRNA to investigate the function of Prdx6 in the protective effects of pretreatment with curcumin on serum from OLV patient-induced lung injury.

First, we transfected A549 cells with Prdx6-siRNA or NC-siRNA and verified its effects by Western blot and qRT–PCR. Second, siRNA-transfected cells were pretreated with 40 μg/ml curcumin for 2 h, and the inflammation and oxidative stress injury model induced by 20% OLV_after_ serum was established, and NC-siRNA was used as a control. Then, inflammatory oxidative stress factors and the production of ROS, as well as NF-κB pathway-related proteins, were detected. Furthermore, the expression of P65 was analyzed by immunofluorescence, and the Proteolytic Nuclear and Cytoplasmic Protein Extraction kit (Cat. No: P5103, NCM) was used to extract cytoplasmic protein and nucleoproteins from the treated cells. In addition, we treated the cells with 5 μM SC75741 (a specific NF-κB signaling pathway inhibitor, MCE, HY-10496) for 48 h to delineate the detailed mechanism.

## Experimental method

### qRT–PCR

An RNA rapid extraction kit (EZBioscience, China) was used to extract total RNA from the treated A549 cells according to the manufacturer’s instructions. The concentration and purity of extracted RNA were analyzed by a Nanodrop 2000 (Thermo Fisher Scientific, USA). cDNA was prepared using the RevertAid First Strand cDNA Synthesis kit (Thermo Fisher Scientific, USA). The qRT–PCR protocol was set as 95 °C for 2 min, followed by 40 cycles of 10 s at 95 °C and 30 s at 60 °C. The primers were designed as follows: IL-6 (forward, 5′-AGACAGCCACTCACCTCTTCAG-3′; reverse, 5′-TTCTGCCAGTGCCTCTTTGCTG-3′); peroxiredoxin 6 (forward, 5′-CAGCTACCACTGGCAGGAACTT-3′; reverse, 5′-GGAAGGACCATCACACTATCCC-3′); and GAPDH (forward, 5′-GTCTCCTCTGACTTCAACAGCG-3′; reverse, 5′-ACCACCCTGTTGCTGTAGCCAA-3′). The expression levels of the target genes were normalized to the levels of GAPDH as an endogenous control in each group. The relative fold expression level of mRNAs was calculated using the 2^−ΔΔCt^ method.

### Immunoblotting

Total protein lysates from cells were loaded on SDS–PAGE gels for electrophoretic separation and transferred onto polyvinyl difluoride membranes, blocked with 5% milk and cut prior to hybridisation with the following primary antibodies: anti-IL-6 (absin; #abs135607; 1:1000), anti-IL-1β (absin; #abs235771; 1:1000), anti-IL-10 (absin; #abs136414; 1:1000), anti-TNF-α (CST; #3707S; 1:1000), anti-Peroxiredoxin 6 (Abcam; #ab133348; 1:1000), anti-phospho-IKKα/β (CST; #2697S; 1:1000), anti-phospho-IκB alpha (Affinity; #AF2002; 1:1000), anti- IκB alpha (Affinity; #AF5002; 1:1000), anti-NF-κB P50 (CST; #3035; 1:1000), anti-NF-κB P65 (CST; #8242; 1:1000), anti-β-Actin (Proteintech; #60008-1-Ig; 1:1000) anti-LaminB1 (CST; #13435; 1:1000) and anti-GAPDH (CST; #2118S; 1:1000). On the second day, the membranes were incubated with HRP-conjugated secondary antibodies (Abcam; goat anti-rabbit IgG, goat anti-mouse IgG; 1:10,000). Subsequently, an enhanced chemiluminescence solution was used to detect the immunocomplexes. The relative quantification of protein was measured using ImageJ software.

### Reactive oxygen species (ROS) assay

ROS generation was evaluated using 2’,7’-dichlorofluorescin diacetate (DCFH-DA, D6883, Sigma–Aldrich, USA) staining. In simple terms, the cells were incubated with 10 μM DCFH-DA for 30 min at 37 °C in the dark. Then, the stained cells were washed three times with PBS. A detected signal with an excitation wavelength of 488 nm and an emission wavelength of 525 nm by a fluorescence microscope (Nikon ECLIPSE Ti2, Nikon, Japan) was used. The fluorescence intensity of DCF-DA was normalized to negative control.

### Enzyme-linked immunosorbent assay (ELISA)

The levels of superoxide dismutase (SOD, MM-0390H2, Meimian Industrial Co., China) and malondialdehyde (MDA, JL11466, J&L Biological, China) in the cell culture supernatant were quantified using ELISA kits. All indices were analyzed by the same technician according to the manufacturer’s instructions.

### Immunofluorescent staining

The cells were washed three times with PBS, fixed with 4% paraformaldehyde and permeabilized with 0.1% Triton X-100 in PBS. After blocking with 5% bovine serum albumin (BSA) in ddH_2_O for 40 min at room temperature, the cells were incubated with a diluted primary antibody (anti-peroxiredoxin 6, Abcam, #ab133348, 1:100; anti-NF-κB P65, CST, #8242, 1:100) at 4 °C overnight, followed by secondary antibody incubation. Cell nuclei were stained with DAPI (Thermo Fisher Scientific, P36966, 20 μg/mL). Representative images were acquired using a confocal microscope image system (OLYMPUS FV1000, OLYMPUS, Japan).

### Statistical analysis

Statistical analyses were conducted using GraphPad Prism 7 (GraphPad Software, Inc., La Jolla, CA, USA) software. Comparisons between the two groups and among multiple groups were performed using two-sided *t-test*s and one-way ANOVA, respectively. All data are expressed as the mean ± standard deviation (SD). A *P value* < 0.05 was considered statistically significant.

## Results

### Twenty percent OLV_after_ serum induced the most obvious inflammation and oxidative stress in A549 cells

**Fig. 1:**
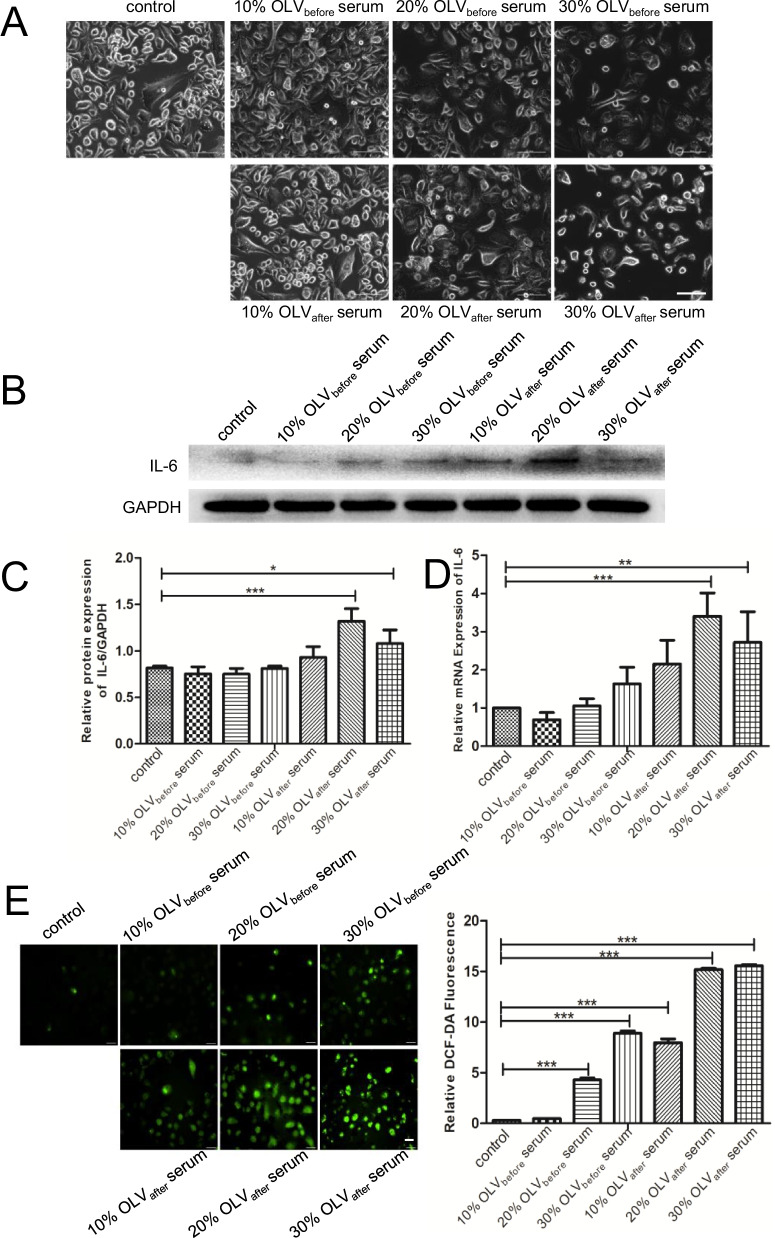
Twenty percent OLV_after_ serum induced the most obvious inflammation and oxidative stress in A549 cells. **A** Representative image of cell morphology after treatment with different concentrations of serum. Scale bar = 100 μm. **B** Western blot analysis images of IL-6 in each group. **C** The relative protein levels of IL-6 were calculated. **D** qRT–PCR analysis of IL-6 in each group.** E** ROS levels were measured by DCFH-DA staining, and the relative DCF-DA fluorescence intensities were normalized and summarized. Scale bar = 100 μm. The results are shown as the means ± SD of 3 individual experiments. *P < 0.05; **P < 0.01; ***P < 0.001

After 48 h of intervention with 10%, 20%, and 30% OLV_before_ serum or OLV_after_ serum in A549 cells, the cell morphology was observed with a microscope in each group. As shown in Fig. 1A, when compared with the control group, in both the OLV_before_ serum and OLV_after_ serum intervention groups, with the increase in serum concentration, the cell density per unit area gradually decreased, while the number of irregular cells gradually increased. Then, the expression of IL-6 at the protein and gene levels was detected by Western blot and qRT–PCR, respectively. The results indicated that 20% OLV_after_ serum could induce the highest level of IL-6 among all the groups, at both the protein and genetic levels, and produces the most obvious inflammatory response in A549 cells (Fig. 1B–D). In addition, the ROS assay showed similar results, which confirmed that 20% OLV_after_ serum could induce a significant oxidative stress response (Fig. 1E).

### Pretreatment with 40 μg/ml curcumin in A549 cells is a suitable concentration to alleviate OLV serum-induced inflammation and oxidative stress

**Fig. 2:**
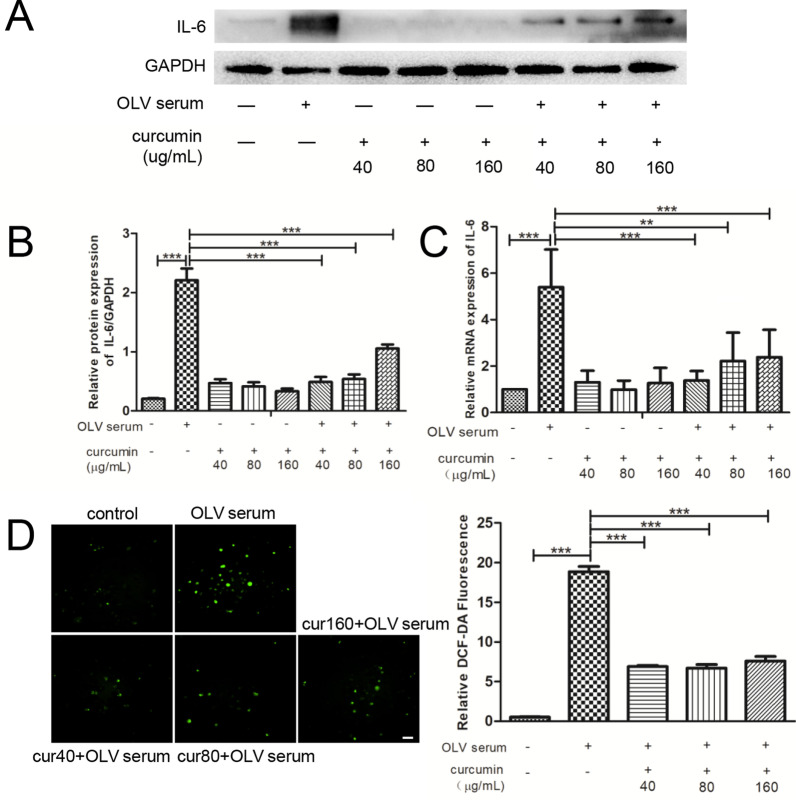
Different concentrations of curcumin alleviate inflammation and oxidative stress in A549 cells. **A** Western blot analysis images of IL-6 in each group. **B** The relative protein levels of IL-6 were calculated. **C** qRT–PCR analysis of IL-6 in each group. **D** ROS levels measured by DCFH-DA staining and the relative DCF-DA fluorescence intensities were normalized and summarized. Scale bar = 50 μm. The results are shown as the means ± SD of 3 individual experiments. *P < 0.05; **P < 0.01; ***P < 0.001

A549 cells were pretreated with different concentrations of curcumin for 2 h, followed by 48 h of 20% OLV_after_ serum intervention. Then, the expression of IL-6 and the level of ROS were assessed. As shown in Fig. 2A–C, 20% OLV_after_ serum significantly increased the level of IL-6 at both the protein and gene levels. Compared with the control group, pretreatment with 40 μg/ml, 80 μg/ml and 160 μg/ml curcumin alone without serum intervention slightly increased IL-6 levels, but no significant difference was observed. Compared with the serum intervention group, pretreatment with curcumin at 40 μg/ml and 80 μg/ml in serum intervention cells reduced the protein level of IL-6 most significantly. In the ROS assay, the results indicated that 20% OLV_after_ serum could cause an obvious oxidative response. After pretreatment with curcumin, the production of ROS decreased significantly, and the reduced level of ROS in the three curcumin treatment groups was similar (Fig. 2D). Based on the above results, to minimize additional drug damage to cells, we chose the lower concentration (40 μg/ml) of curcumin for the following experiments.

### Curcumin pretreatment reduced OLV serum-induced inflammation and oxidative stress and could be related to Prdx6

**Fig. 3:**
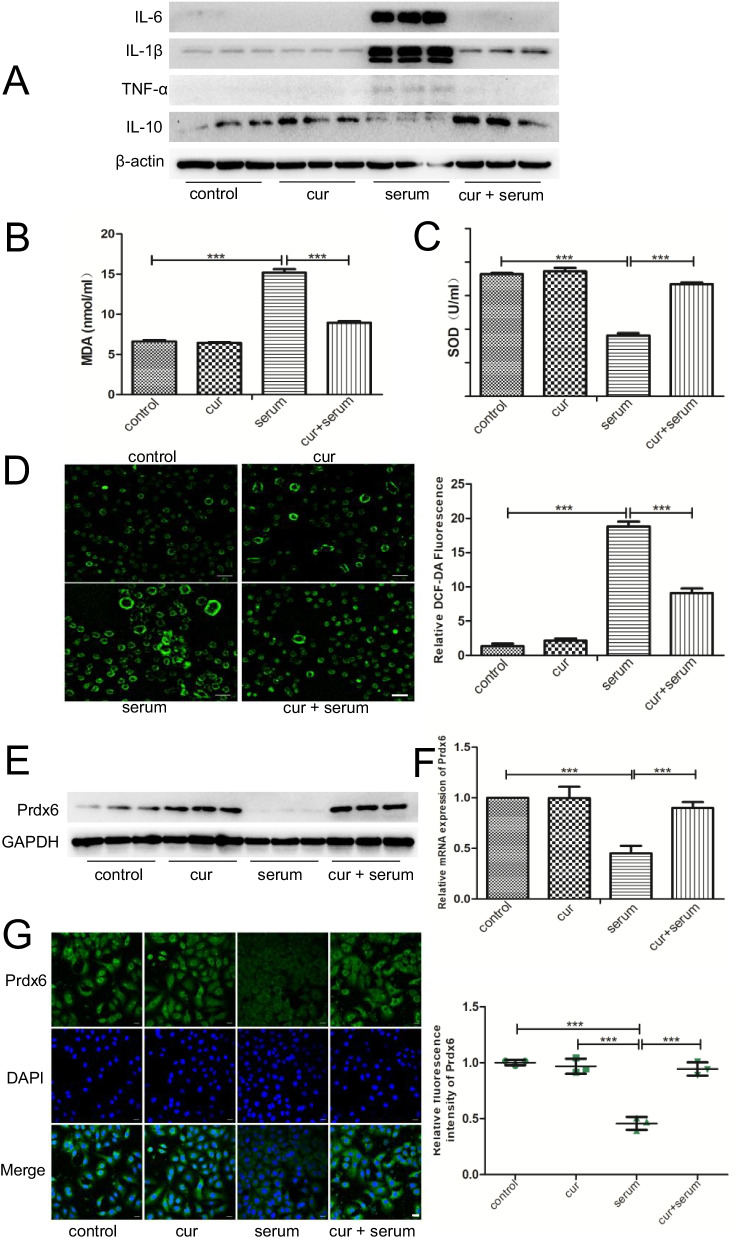
The protective effect of curcumin on OLV-induced ALI is related to Prdx6. **A** Western blot analysis of representative inflammatory factor levels, among which IL-6, IL-1β, and TNF-α are the classic proinflammatory factors and IL-10 is the anti-inflammatory factor in each group. **B** ELISA analysis of MDA levels in each group. **C** ELISA analysis of SOD levels in each group. **D** ROS levels were measured by DCFH-DA staining in each group. Scale bar = 100 μm. **E** Western blot analysis images of Prdx6 in each group. **F** qRT–PCR analysis of Prdx6 in each group. **G** Immunofluorescence analysis of Prdx6 in each group. DAPI is shown in blue and represents the nucleus. Scale bar = 100 μm. In the control group (control): cells were pretreated with the DMSO vehicle alone for 2 h, then changed to a serum-free medium for 48 h; in the curcumin group (cur): cells were pretreated with 40 μg/ml curcumin + DMSO for 2 h, then changed to serum-free medium for 48 h; in the serum group (serum): the cells were intervened with 20% OLV_after_ serum for 48 h; in the curcumin + serum group (cur + serum): the cells were first pretreated with 40 μg/ml curcumin and DMSO, then changed to 20% OLV_after_ serum for 48 h. The results are shown as the means ± SD of 3 individual experiments. *P < 0.05; **P < 0.01; ***P < 0.001

Pretreatment with 40 μg/ml curcumin was used to intervene in the inflammation and oxidative stress induced by 20% OLV_after_ serum in A549 cells and to explore the effect of curcumin on the injury induced by serum collected from patients undergoing OLV in alveolar epithelial cells. We detected the protein expression of classic proinflammatory and anti-inflammatory cytokines, such as IL-6, IL-1β, TNF-α and IL-10, by Western blot. At the same time, the expression of MDA and SOD in the cell culture supernatant was quantified using an ELISA kit. As shown in Fig. 3A–C, serum from patients undergoing OLV stimulation produced obvious inflammatory and oxidative reactions, mainly manifested by an increase in expressions of IL-6, IL-1β, TNF-α and MDA and a decrease in expressions of IL-10 and SOD. Pretreatment with curcumin reversed these changes. Similarly, the results of the ROS assay also confirmed that curcumin could significantly reduce the increase in ROS content induced by OLV serum (Fig. 3D). Furthermore, we observed the expression of Prdx6 in each group by Western blot, qRT–PCR and immunofluorescence and found that OLV serum could significantly reduce the expression of Prdx6, while pretreatment with curcumin could reverse it (Fig. 3E–G).

### Prdx6 in the protective effects of curcumin pretreatment on serum from OLV patient-induced injury and association with the NF-κB pathway

**Fig. 4:**
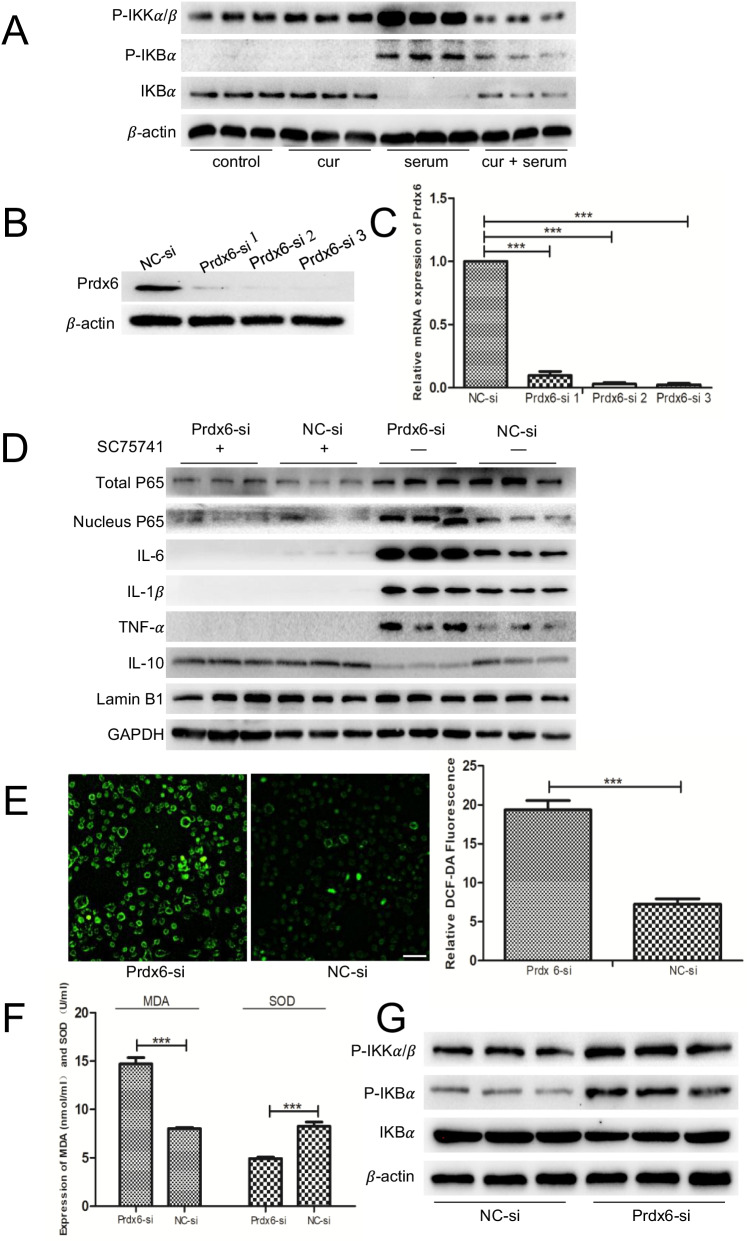
Prdx6 mediated the protective function of curcumin in OLV-induced ALI by regulating the NF-κB pathway. **A** The levels of NF-κB signaling markers in each group were determined via Western blot. **B** Western blot analysis of Prdx6 expression in A549 cells transfected with either Prdx6-siRNA or NC-siRNA. **C** qRT–PCR analysis of Prdx6 expression in A549 cells transfected with either Prdx6-siRNA or NC-siRNA. **D** The expression of representative inflammatory factors and P65 in Prdx6-siRNA- or NC-siRNA-transfected cells with or without SC75741. **E** ROS levels were measured by DCFH-DA staining in cells transfected with either Prdx6-siRNA or NC-siRNA. Scale bar = 100 μm. **F** ELISA analysis of oxidative stress factor levels (MDA and SOD) in cells transfected with either Prdx6-siRNA or NC-siRNA. **G** The protein levels of NF-κB signaling markers in cells transfected with either Prdx6-siRNA or NC-siRNA. In the control group (control): the cells were pretreated with the DMSO vehicle alone for 2 h, then changed to a serum-free medium for 48 h; curcumin group (cur): the cells were pretreated with 40 μg/ml curcumin + DMSO for 2 h, then changed to serum-free medium for 48 h; serum group (serum): the cells were intervened with 20% OLV_after_ serum for 48 h; curcumin + serum group (cur + serum): the cells were first pretreated with 40 μg/ml curcumin and DMSO, then changed to 20% OLV_after_ serum for 48 h. The results are shown as the means ± SD of 3 individual experiments. *P < 0.05; **P < 0.01; ***P < 0.001

To explore the potential mechanism of the protective effect of curcumin, we examined the proteins involved in the NF-κB pathway. After OLV serum intervention, the expression of P-IKKα/β and P-IKBα was increased significantly, while that of IKBα was decreased. When curcumin was used, the expression of P-IKKα/β and P-IKBα was significantly decreased, while that of IKBα was increased (Fig. 4A). To verify the relationship between Prdx6 and the NF-κB signaling pathway, we transfected A549 cells with Prdx6-siRNA or NC-siRNA. As shown in Fig. 4B, C, Prdx6-siRNA significantly reduced the expression of Prdx6 at both the protein and gene levels. Among them, Prdx6-siRNA 2 and siRNA 3 possessed the most obvious reduction effect. Therefore, we used Prdx6-siRNA 2 or siRNA 3 to transfect A549 cells. The transfected cells were pretreated with 40 μg/ml curcumin for 2 h, and the inflammation and oxidative stress injury model induced by 20% OLV_after_ serum was established. Compared with that in the NC-siRNA group, the expression of pro-inflammatory factors, including IL-6, IL-1β and TNF-α, in the Prdx6-siRNA group was significantly increased and anti-inflammatory IL-10 markedly reduced (Fig. 4D). ROS assays demonstrated that ROS generation in the Prdx6-siRNA group was significantly higher than that in the NC-siRNA group (Fig. 4E). Our ELISA results further confirmed that when compared with the NC-siRNA group, the expression of the pro-oxidative stress factor MDA significantly increased, while the expression of the antioxidative stress factor SOD decreased in the Prdx6-siRNA group (Fig. 4F). Subsequently, we detected NF-κB pathway-related proteins in these two groups and found that compared with the NC-siRNA group, the expression of P-IKKα/β and P-IKBα was significantly increased, while the expression of IKBα was slightly decreased in the Prdx6-siRNA group (Fig. 4G).

### Prdx6 inhibits activation of the NF-κB signaling pathway by suppressing the nucleus translocation of P65 to participate in curcumin pretreatment on serum from OLV patient-induced inflammatory damage

**Fig. 5:**
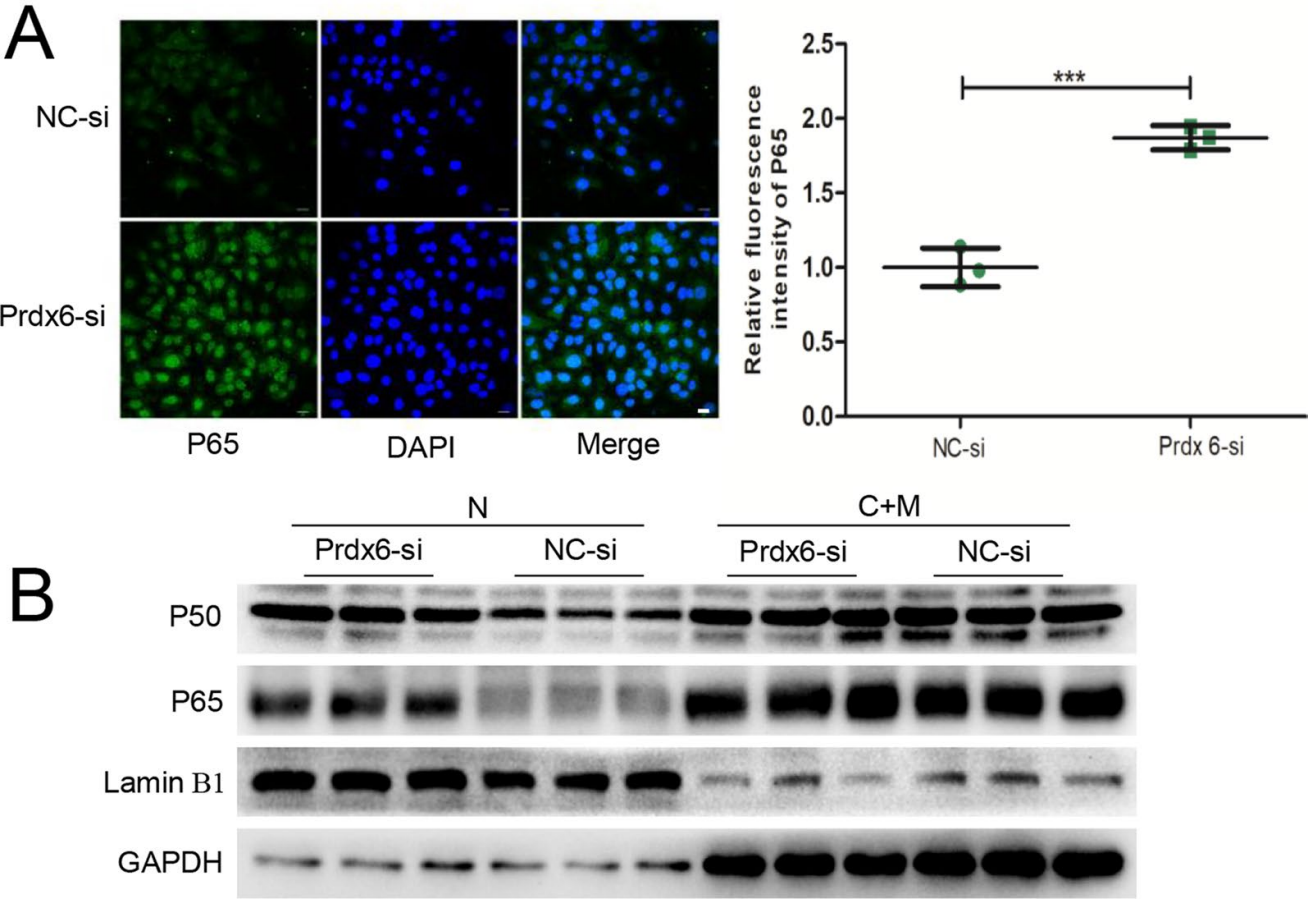
Prdx6 inhibits activation of the NF-κB signaling pathway by suppressing the nuclear translocation of P65. **A** Immunofluorescence analysis of P65 in cells transfected with either Prdx6-siRNA or NC-siRNA. DAPI is shown in blue and represents the nucleus. Scale bar = 100 μm. **B** Nuclear (N) and cytosolic/membrane (C + M) proteins of the NF-κB pathway in Prdx6-siRNA- or NC-siRNA-transfected cells. The cells in all groups were first pretreated with 40 μg/ml curcumin and DMSO and then changed to 20% OLV_after_ serum for 48 h. The results are shown as the means ± SD of 3 individual experiments. *P < 0.05; **P < 0.01; ***P < 0.001

NC-siRNA- or Prdx6-siRNA-transfected A549 cells were pretreated with 40 μg/ml curcumin for 2 h, and the inflammation and oxidative stress injury model induced by 20% OLV_after_ serum was established. Immunofluorescence staining was performed, and the ProtLytic Nuclear and Cytoplasmic Protein Extraction kit were used to extract the cytoplasmic protein and nucleoprotein of the two groups to detect P65 expression in the treated cells. The results of the two detection methods in Fig. 5A, B showed that P65 was in both the cytoplasm and nucleus of curcumin-pretreated A549 cells. Additionally, compared to the NC-siRNA group, the expressions of P50 and P65 in the nucleus of the Prdx6-siRNA group were significantly higher, suggesting that Prdx6 suppressed P65 and P50 translocation into the nucleus to inhibit activation of the NF-κB signaling pathway. To further confirm the role of the NF-κB signaling pathway in the protective effect of curcumin pretreatment on serum from OLV patient-induced inflammatory damage, we performed a rescue assay using SC75741, which is a specific NF-κB inhibitor. As shown in Fig. 4D, SC75741 attenuated inflammatory damage induced by 20% OLV_after_ serum in A549 cells, as determined by inhibiting the expression of proinflammatory factors such as IL-6, IL-1β and TNF-α and increasing the levels of anti-inflammatory IL-10.

In summary, curcumin preconditioning exerted a protective effect on lung injury induced by serum from OLV patients in A549 cells by increasing the expression of Prdx6. On the one hand, Prdx6 can reduce the inflammatory response by suppressing the nuclear translocation of P65 to inhibit activation of the NF-κB signaling pathway, and on the other hand, Prdx6 can also reduce oxidative stress injury by increasing the activity of antioxidant enzymes and inhibiting the activity of pro-oxidation factors.

## Discussion

In this study, we simulated acute lung injury caused by OLV at the cellular level for the first time, which can be used in future relevant research models. Here, we showed that the application of 20% OLV_after_ serum could induce obvious inflammation and oxidative stress in A549 cells. We further found that 40 μg/ml curcumin pretreatment could significantly alleviate OLV serum-induced inflammation and oxidative stress and was related to Prdx6 regulation.

As a special mode of mechanical ventilation, OLV is a highly complex procedure and plays a key role, especially in thoracic surgery. However, like a double-edged sword, it can also be harmful to patients due to ventilator-induced lung injury caused by various mechanisms [20]. During the period of OLV in lung surgery, despite the normalization of hemodynamic and ventilatory patterns, the ventilation/perfusion (V/Q) mismatch still exists in both the dependent and non-dependent lungs, which has more damaging consequences than does a period of complete lung collapse and surgical manipulation [21]. In addition, according to the “multiple-hit hypothesis,” some researchers regard lung injury caused by two-lung ventilation in thoracic surgery as the first hit. The second hit is caused by OLV and surgical procedures. Alveolar recruitment and accompanying re-expansion/reperfusion-induced lung injury are considered the third hit [22]. Multiple damage factors work together to induce lung injury, characterized by diffuse epithelial and endothelial injury with infiltration of inflammatory cells, recruitment of leukocytes to the lung and increased lung permeability, resulting in lung edema, surfactant dysfunction and deterioration of pulmonary gas exchange. Proinflammatory cytokines such as IL-6, IL-1β and TNF-α play crucial roles in inflammation. Among them, IL-6 is a major player in cytokine storms, and increased levels of IL-6 are associated with the severity of acute inflammation. In addition, the serum levels of IL-6 and TNF-α are known to be significant predictors of disease severity and death [23, 24]. Therefore, IL-6 was selected as an indicator of the inflammatory response in our study. Moreover, the lungs are major organs of systemic and pulmonary oxidative stress. Clinical and experimental studies have shown that OLV in thoracic surgery is a powerful free radical generator [25, 26]. The excessive production of ROS has been proven to be related to the decline of the antioxidant defense system during aging and/or the decline of the antioxidant defense system in cells facing environmental or cellular stress, failing intracellular homeostasis [27]. Oxidative stress caused by this redox imbalance leads to the pathogenesis of a variety of lung diseases, such as ALI and acute respiratory distress syndrome (ARDS) [28, 29]. MDA is a good indicator of free radical activity, and its elevation represents increased lipid peroxidation. SOD is an endogenous antioxidant enzyme that can detoxify free radicals. According to our results, 20% OLV_after_ serum from thoracic surgery patients could induce a significant inflammation and oxidative stress response in vitro, which is characterized by an obvious increase in IL-6 and ROS expressions.

Recently, curcumin has attracted much attention due to its anti-inflammatory, antioxidant, antitumor and other pharmacological effects. Many studies have revealed that curcumin has a protective effect by increasing the expression of endogenous Prdx6 [30–32]. Prdx6 is a 1-cysteine PRDX, a multifunctional enzyme with not only Gpx and aiPLA2 but also LPCAT activity. Prdx6 may reduce aggregate-induced oxidative toxicity via its Gpx activity; in turn, it is possible to regulate inflammation/immune networks and mitochondrial oxidative stress by interacting with pathogenic genes and encouraging toxic protein aggregation through its aiPLA2 activity [33]. Prdx6 is closely related to the occurrence and development of ALI, and it has been shown that Prdx6 deficiency initiates ROS-induced endoplasmic reticulum stress that leads to cell death [34]. Fisher’s team found that the inactivation of peroxiredoxin 6 phospholipase A2 activity can protect against lung injury in mouse models of ventilator-induced lung injury and LPS-induced ALI [18, 35]. Moreover, many studies have shown that Prdx6 knockout aggravates LPS-, cecal ligation- and puncture-induced ALI by augmenting inflammation, oxidative stress and matrix degradation [18, 36]. In our research, 20% OLV_after_ serum decreased the expression of Prdx6 in A549 cells, and curcumin pretreatment not only obviously reversed the expression of Prdx6 but also reduced the increased levels of inflammatory factors and reactive oxygen free radicals to reduce inflammation and oxidative stress injury, which was consistent with results reported in other studies [30, 31]. Subsequently, we used siRNA to verify the relationship between Prdx6 and lung injury and found that Prdx6-siRNA aggravated inflammation and oxidative stress induced by serum from patients undergoing OLV. Our results suggest that Prdx6 is essential for cellular protection. A deficiency in Prdx6 not only increased the production of proinflammatory factors and oxygen free radicals but also decreased the expression of anti-inflammatory factors and antioxidant enzymes. In our model of lung injury induced by OLV in vitro, curcumin pretreatment inhibited inflammation and oxidative stress to protect cells by stimulating endogenous Prdx6 production.

Nuclear factor-kappa B (NF-κB) signaling regulates important physiological processes, such as inflammation, immune responses, cell survival and cancer. When cells are stimulated, the IKK complex (IKKα, IKKβ and IKKγ) in the cytoplasm is rapidly activated by proinflammatory signaling cascades, and then IKBα is phosphorylated and recognized by ubiquitinating enzymes. Subsequently, the resulting proteasomal degradation of IκB proteins releases IκB-bound NF-κB transcription factors (P50 and P65), which are transferred to the nucleus to drive the expression of target genes [37]. There is evidence that Prdx6 plays its biological role by regulating the NF-κB signaling pathway [31, 38, 39]. Prdx6 interrupts the formation of the TRAF6-ECSIT complex induced by TLR4 stimulation, leading to the suppression of bactericidal activity by inhibiting NF-κB activation in the cytoplasm [39]. In addition, Prdx6 also plays a protective role against radiation by interacting with the TLR4 receptor and subsequently activating the NF-κB signaling pathway to trigger cellular defense mechanisms [38]. In the present study, curcumin pretreatment restored Prdx6 downregulation, which in turn normalized NF-κB signaling. Further application of SC75741, an inhibitor of NF-κB, reduced the effect of Prdx6-siRNA on the OLV serum-induced inflammatory response, suggesting the involvement of the NF-κB signaling pathway in the protective effects of Prdx6. As with our results, Chhunchha et al. found that curcumin could attenuate endoplasmic reticulum stress and NF-κB-mediated abnormal signaling to reduce hypoxia-induced oxidative stress-mediated cell death in the mouse hippocampal cells (HT22) by increasing Prdx6 expression [31]. We further investigated the specific mechanism by which Prdx6 regulates the NF-κB signaling pathway by detecting the expression of nuclear P65 and P50 and found that the expression levels of P65 and P50 in the Prdx6-siRNA group were higher than those in the NC-siRNA group, indicating that Prdx6 could suppress the nuclear translocation of P65 and P50. Our immunofluorescence results further confirmed these findings. However, further studies are required to clarify the mutual regulatory mechanism between Prdx6 and the NF-κB signaling pathway.

There are several limitations to our study. First of all, we collected serum from patients with OLV to simulate OLV-induced systemic inflammation, however, since the potential mechanisms of ALI induced by OLV are complex and intertwined, the stimulating factors contained in the blood cannot fully represent the injury caused by OLV in thoracic surgery patients. Second, as a complex system, the body will have a series of responses to non-physiological operations such as OLV and surgery. It is a systematic response, and A549 cells could not replace the body’s response. Then, although the A549 cell line has some characteristics of alveolar type II epithelial cells, it is generally used as the carrier of the acute lung injury cell model [40, 41]. However, its essence is still a non-small cell lung cancer cell line, which cannot completely replace normal lung tissue cells. Finally, our study confirms that curcumin pretreatment reduces ALI induced by OLV significantly in vitro. Clinical medication is usually therapeutic. Although curcumin pretreatment has obvious anti-inflammatory and antioxidant effects, it is still different from clinical drug application methods. This is another limitation of this study, which we need to consider and improve in our next research.

## Conclusions

In our inflammation and oxidative stress injury model induced by 20% OLV_after_ serum in vitro, downregulation of Prdx6 led to the activation of the NF-κB signaling pathway, which caused the subsequent overproduction of inflammatory cytokines and ROS. Pretreatment with curcumin restored Prdx6 downregulation and inhibited NF-κB pathway activation by suppressing the nuclear translocation of P65 and P50. Curcumin protected against ALI not only by reducing the expression of proinflammatory and pro-oxidative factors but also by increasing the expression of anti-inflammatory and antioxidative factors. All these factors eventually reduce inflammation and oxidative stress damage induced by serum from patients undergoing OLV with thoracic surgery. Our findings offer new insights into the mechanism of ALI induced by OLV, and the protective effect of curcumin provides a new reference for its prevention and treatment.

## Data Availability

The datasets used and/or analyzed during the current study are available from the corresponding author on reasonable request.

## Abbreviations

OLV: One-lung ventilation
ALI: Acute lung injury
ARDS: Acute respiratory distress syndrome
Prdx6: Peroxiredoxin 6
NF-κB: Nuclear factor kappa B
Gpx: Glutathione peroxidase
aiPLA2: Calcium-independent phospholipase A2
LPCAT: Lysophosphatidylcholine acyltransferase
FBS: Fetal bovine serum
BSA: Bovine serum albumin
LPS: Lipopolysaccharide
VATS: Video-assisted thoracoscopic surgery
ROS: Reactive oxygen species
SOD: Superoxide dismutase
MDA: Malondialdehyde
SpO_2_: Pulse oxygen saturation
V/Q: Ventilation/perfusion
qRT–PCR: Quantitative reverse transcription PCR
ELISA: Enzyme-linked immunosorbent assay
DCFH-DA: Dichlorodihydrofluorescein diacetate
